# Evaluation of a prison violence prevention program: impacts on violent and non-violent prison infractions

**DOI:** 10.1186/s40621-023-00450-9

**Published:** 2023-07-24

**Authors:** Molly Remch, Gregory Swink, Charles Mautz, Anna E. Austin, Rebecca B. Naumann

**Affiliations:** 1 Department of Epidemiology, Gillings School of Global Public Health, University of North Carolina at Chapel Hill, 2101 McGavran-Greenberg Hall, CB #7435, Chapel Hill, NC 27599-7435 USA; 2 North Carolina Department of Adult Correction (Formerly Named North Carolina Department of Public Safety at Time of Analysis), Raleigh, NC USA; 3 Department of Maternal and Child Health, Gillings School of Global Public Health, University of North Carolina, Chapel Hill, NC USA; 4 Injury Prevention Research Center, University of North Carolina, Chapel Hill, NC USA

**Keywords:** Prisons, Violence prevention, Violence intervention, Behavioral infractions, Solitary confinement, Substance use, Diversion

## Abstract

**Background:**

Individuals who commit acts of violence in prisons are often placed in highly controlled environments called restrictive housing (i.e., solitary confinement), which can have severe physical and mental health consequences and does not reduce violence. As such, North Carolina prisons have introduced the rehabilitative diversion unit (RDU) to reduce the use of restrictive housing and reduce violence in prison.

**Methods:**

We evaluated the effect of the RDU on prison infractions. We compared rates of infractions by type (including violent infractions) among men enrolled in the RDU and men who were eligible for the RDU but placed in restrictive housing for control purposes (RHCP). We also evaluated sustained program impacts by comparing the hazard of first infraction among these same two groups of men after program completion, when they had returned to the general prison population. Finally, we compared the hazard of first promotion to a less restrictive custody level (medium custody) when these men had returned to the general prison population.

**Results:**

The primary analytic cohort was made up of 3128 men contributing 897,822 person-days. Adjusted rates of violent infractions were lower in the RDU than in RHCP (adjusted rate ratio: 0.6; 95% CI: 0.4, 1.1). All other categories of infractions, including drug-related infractions, occurred at higher rates during RDU, as compared to RHCP. In analyses of sustained program impacts, for most categories of infractions, there were no differences in the hazard of first infraction post-RDU and post-RHCP. However, the hazard of violent infraction post-RDU was higher (adjusted hazard ratio: 2.1; 95% CI: 1.1, 4.0) than post-RHCP. The hazard of promotion to a less restrictive custody level was higher post-RDU (adjusted hazard ratio: 17.4; 95% CI: 7.2, 42.2) than post-RHCP.

**Conclusions:**

We found the RDU program may be effective in reducing violence for men enrolled in the program, but that these benefits were not sustained. Continued programming may be a useful tool to transition men from the programmatically intensive environment of the RDU to the general prison population. Additionally, we recommend the expansion of evidence-based treatment for substance use disorder.

**Supplementary Information:**

The online version contains supplementary material available at 10.1186/s40621-023-00450-9.

## Background

Violence is a leading cause of death in the USA, particularly among individuals under the age of 34 years (Centers for Disease Control and Prevention, n.d.), and contributes to negative long-term mental, physical, social, emotional, and economic outcomes (Rivara et al. 2019). Critical drivers of violence perpetration occur at the individual, relational, community, and societal levels and include poor impulse and behavior control, family economic stress and conflict, exposure to neighborhood violence, and income inequality (Decker et al. 2018). Prisons are a high-risk environment for violence, given the confluence of individual (e.g., mental health and behavioral disorders) (Schenk and Fremouw 2012), relational (e.g., history of trauma), and community or environmental risk factors (e.g., prison culture as an inherently dangerous and stressful environment) (Blitz et al. 2008; Fazel et al. 2016; Rocheleau 2015; Wooldredge 2020).

Prisons often attempt to prevent acts of violence by placing individuals in different levels of custody, or security, based on past behavior, perceived threat, or likelihood of disrupting prison order and safety. In North Carolina (NC), prison custody levels include close, medium, and minimum, with close and medium level assignments generally given to individuals perceived to be a higher risk to prison safety (North Carolina Department of Public Safety, n.d.-a). Research indicates that more restrictive (i.e., higher levels of) custody is associated with mortality following release from prison (Bukten et al. 2022), recidivism (Gaes and Camp 2009), and negative mental health outcomes (Grassian 1983; Haney 2003; Miller and Young 1997; Smith 2006).

Individuals who commit violent acts or other infractions in prison are typically placed in an even more restricted environment, called restrictive housing (referred to by some as solitary confinement). In NC state prisons, individuals that have committed violent acts are commonly placed in a type of restrictive housing called Restrictive Housing for Control Purposes (RHCP), a long-term assignment used to control behaviors of incarcerated persons posing repeated disruption, threats to the safety of staff or others, or threats to safe and secure facility operations. Other types of restrictive housing used in NC state prisons include Restrictive Housing for Administrative Purposes (RHAP) and Restrictive Housing for Disciplinary Purposes (RHDP), which generally represent shorter term restrictive housing assignments for disciplinary decision-making and less severe infractions, respectively. While considerable variation exists in RHCP assignment length, the average RHCP assignment exceeds 5 months. Despite multiple assignment types within restrictive housing, any such assignment restricts the individual to their cell for 22 or more hours each day. Time outside of the cell may involve exercise indoors and/or outdoors, non-contact visitations, healthcare appointments, group therapy or educational opportunities, or other common personal needs (e.g., phone calls, showers, meetings with case managers, etc.). Property retained in-cell is restricted; however, individuals are generally allowed typical clothing, personal items, approved religious materials, books, letters and addresses, hygiene products, and various other day-to-day items. NC DAC protocols stipulate multiple levels of supervisory review within and/or outside (i.e., regional, central office) for more lengthy assignments to restrictive housing, whereas brief lengths of stay may be enacted at the discretion of unit-level or facility-level staff when deemed warranted.

Long-term restrictive housing assignments, such as RHCP, are not associated with reductions in prison violence and are demonstrated to be associated with poor individual health and wellbeing outcomes. Existing studies indicate that long-term restrictive housing does not affect the likelihood of future misconduct among those with an initial violent offense, both during incarceration (Labrecque and Smith 2019; Medrano et al. 2017; Morris 2016) and after prison-release (James and Vanko 2021; Luigi et al. 2022). Prior research also shows that long-term restrictive housing is associated with serious psychological and physiological damage, sustained well beyond the episode of restrictive housing or even incarceration (James and Vanko 2021). Psychological harms include suicidality, self-harm, anxiety, depression, loss of identity, hyperresponsivity, and post-traumatic stress disorder (PTSD) (James and Vanko 2021; Luigi et al. 2020). Physiological impacts include premature death, including from opioid overdose, hypertension, and heart attacks (Luigi et al. 2020; Strong et al. 2020). However, a longitudinal study out of the Colorado Department of Corrections did not find administrative segregation, the local term for restrictive housing, to be associated with mental health deterioration (O’keefe et al. 2011). Thus, more research is needed in this area.

Given the demonstrated detrimental associations of highly restrictive prison environments, including long-term restrictive housing, with individual health, and the need for a structured environment with rehabilitative services for individuals committing acts of violence prior to transition to a general prison population, the NC Department of Adult Correction (DAC) created the rehabilitative diversion unit (RDU). At the time, this research was conducted, NC prisons were operated by the NC Department of Public Safety; however, prisons are now operated by the newly created NC Department of Adult Correction, independent of the Department of Public Safety. We refer to this agency throughout as the Department of Adult Correction.

The RDU program, in operation with approximately a 500-participant capacity in Marion Correctional Facility since 2016, is intended to reduce prison violence among individuals who would otherwise be assigned to RHCP, as well as provide a structured rehabilitative programming-oriented pathway to safe return to the general prison population. The 12–18-month program, available to adult men at least 21 years old, is divided into three phases. In the first two phases, the program focuses on providing skills and tools to improve emotional and psychological health, physical well-being, social skill development, and relationship building. The third phase of the program includes a focus on educational coursework (e.g., preparation for the HiSET, a high school equivalency test). When men enter the RDU, the environment is similar to RHCP. Progression through the program is based on assignment completion, overall participation, and reduction in negative behaviors. As participants progress, out of cell time and other privileges increase. RDU staff, comprised of select correctional officers and rehabilitative service staff, are trained on psychotherapeutic skills, such as motivational interviewing, crisis intervention, and cognitive behavioral intervention. As space is limited to enter the RDU at any given time, participants are selected from a pool of RDU-eligible men by RDU administrative staff at the direction of the RDU Coordinator. Program eligibility includes, among other factors, having a history of infractions resulting in current RHCP assignment, with priority placement given to men with a history of more serious infractions, particularly those with a history of violent infractions. Each week, RDU staff review lists of men currently assigned to RHCP to identify any RDU-eligible men. A list of RDU-eligible men is then reviewed by medical staff, mental health staff, and the RDU coordinator to ensure they are appropriate fits for the RDU. Additional details on RDU eligibility criteria and selection processes are described below in the “Methods” section.

Completion times of each phase and thus the overall RDU program vary depending on individual engagement, disciplinary issues, or lack thereof. That is, participants can progress at an individualized pace through each step of the program, as defined by their completion of required programmatic elements and requisite approval from RDU staff. Staff share responsibilities of providing and facilitating programming elements; thus, RDU participants commonly engage with various staff throughout their experience. In general, those who chose not to participate or unfortunately incur disciplinary issues may be placed in a non-participating status, conditions of which mimic restrictive housing. Varied efforts are made by RDU and facility staff to transition these men back into RDU participation as quickly, safely, and equitably as possible. Program completion is generally attained by completion of all required programmatic materials and safe adjustment to unrestrained, group activities.

Given the need for programming that reduces violence in prisons and removes exposure to restrictive housing environments, the purpose of this analysis was to evaluate and assess the impact of the RDU on critical safety-related outcomes. Specifically, we aimed to examine the impact of the RDU, as compared to RHCP (i.e., the standard placement for individuals with violent infractions), on future violent and non-violent infractions, both while in the program and following program release back into the general prison population. Additionally, given prior research on harmful impacts of restrictive prison environments, we also investigated the impact of the RDU program on custody-level changes following release from the RDU or RHCP assignment and while back in the general prison population.

## Methods

To examine the impact of the RDU, as compared to RHCP, on infractions and custody level, we conducted an observational cohort study. To understand the effect of the RDU, we examined outcomes during two different time periods. First, we compared rates of infractions during RDU placement to rates of infractions during RHCP placement. Second, we compared the time to first infraction and first change in custody level following RDU or RHCP completion, when participants had returned to the general prison population.

### Data

We used administrative data from the NC DAC. We received data on all incarcerations for all adults released from NC DAC prisons between 2000 and 2020, as well as all individuals who were admitted to the RDU.

### Cohort construction

We constructed a primary analytic sample of individuals who met the RDU eligibility criteria and were either enrolled in the RDU or placed in RHCP, between June 2016, when the RDU began, through February 2020, prior to COVID-19 disruptions and changes in prison programming. Consistent with NC DAC-defined RDU eligibility criteria, we defined RDU eligibility as: an adult male age 21 years or older with a mental health grade of 1 or 2, an IQ score of 75 or higher, and a reading score of 3.8 or higher on the Wide Range Achievement Test 4 (WRAT-4). Mental health is graded on a five-point scale, with higher grades indicating a higher level of treatment services provided (e.g., psychiatric medication, residential or inpatient care, etc.). Mental health grades of 1 or 2 indicate no current behavioral health treatment or current outpatient behavioral health care but no psychiatric care, respectively.

Men who met these criteria at the time of RDU enrollment contributed 417,343 person-days to the RDU-exposed group. Men who met these criteria while placed in RHCP, and who had not been previously enrolled in RDU or another restrictive housing diversion program (i.e., Therapeutic Diversion Units (TDUs, which have also previously been evaluated)) (Remch et al. 2021, 2022), contributed 480,479 person-days to the RHCP-exposed group. Men who contributed person-days to the RHCP-exposed group and later enrolled in the RDU could contribute person-time to both groups. While men could contribute person-time during multiple eligible RHCP stays, they could only contribute person-time up through and including their first RDU stay. Thus, in our analytic sample, men could not move in and out of the RDU.

For our second analysis focused on examining time to first infraction and first custody level change following release from RDU or RHCP assignment, we restricted our sample to men who, after RDU or RHCP assignment completion, were returned directly to the general prison population (i.e., not released from prison, transferred to modified housing, placed in TDU, or, in the case of the RHCP-exposed group, placed directly into the RDU with no time spent in the general prison population). We followed their person-time while they were in the general prison population.

### Measures

*Infractions* We assessed all infractions adjudicated by the internal NC DAC review processes as “guilty,” as well as the following subcategories of infractions: A-level, B-level, C-level, violent, alt-violent, and drug-related.

NC DAC assigns all infractions a code, with a prefix of A, B, or C (North Carolina Department of Public Safety, n.d.-b). These roughly translate to the severity of the infraction, as determined by NC DAC, and the severity of the sanction. A-level infractions are the most severe and include involvement with a gang, possession of a weapon to aid in assault, insurrection, riot, setting a fire, assaulting staff, substance possession, and sexual acts. B-level infractions include disobeying an order, lock tampering, use of profane language, and threatening to harm staff. C-level infractions include unauthorized use of mail, possession of contraband not intended for escape or violence, creating an offensive condition, or bartering or loaning money.

The violent infractions category used in this analysis is not an official NC DAC designation, but as violence reduction is a main goal of the RDU, we include it as an outcome. For this analysis, violent infractions include, but are not limited to, assaulting staff, engaging in a riot, or assaulting a person; these are largely a subset of A-level infractions. We developed the alt-violent infractions category, which is also not an official NC DAC designation, as infractions that have the potential to lead to or are otherwise indicative of violence. Alt-violent infractions are largely a subset of B-level infractions and include threatening staff, assaulting staff in a manner in which injury is unlikely, and weapon possession. Drug-related infractions include substance possession or refusal to submit to a drug or breathalyzer test, both of which are also A-level infractions.

*Custody level* Incarcerated individuals are assigned to one of three custody levels: close, medium, and minimum. Individuals classified as close custody are housed in higher security facilities, with medium and minimum custody allowing successively more privileges and freedom of movement. Only minimum custody allows access to the public via approved work release, study release, or similar programs. Custody level is determined by NC DAC staff through a multi-step review process involving the use of a standardized classification tool including individual static and dynamic factors, as well as multiple layers of supervisory and leadership approval. Typically, custody levels are reviewed no more frequently than every six months and determine the facility housing locations eligible for each individual. By extension, custody assignments influence program eligibility as not all programs are offered uniformly across all prison facilities. Consistent with common prison management practices, custody level partially reflects anticipated safety concerns or risk of violence, with close custody providing the most secure housing environments (e.g., single cell housing, cohort-style movement, limited access to tools or equipment, etc.). Medium custody allows for greater mobility in larger groups, an expansion in possible programming opportunities, and dorm-style housing. Minimum custody expands these characteristics further.

In the RDU or RHCP, individuals are in a close custody environment. Thus, we examined change in custody level only in our second set of analyses. In these analyses, we compared time to first change in custody level for RDU-exposed and RHCP-exposed individuals during return to the general prison population.

### Analytic methods

We calculated the prevalence of demographic and incarceration-related characteristics of individuals included in our primary analytic cohort stratified by person-time contributed during RDU enrollment and RHCP placement.

For our first analysis, we conducted Poisson regression to calculate unadjusted and adjusted rate ratios (RRs) estimating the association of RDU enrollment, as compared to RHCP placement, with infractions while participants were in these assignments. We used a generalized estimating equations approach to account for the correlation within individuals who contributed person-time during multiple exposure periods.

For our second analysis, we used Fine-Gray survival models to calculate unadjusted and adjusted subdistribution hazard ratios (HRs) for the first occurrence of an infraction (overall and by specific infraction type) and custody level change following release from the RDU or RHCP and return to the general prison population. We focused on outcomes occurring within 30 days post-RDU or post-RHCP. Individuals were censored at the first of: 30 days in the general prison population following release from the RDU or RHCP, February 29th, 2020 (the end of the study period), death, or release from prison. We considered the following to be competing events: restrictive housing entry, TDU entry, modified housing entry, RDU entry, and inpatient mental health treatment unit admission. We did not include time spent in these settings because the opportunity for infractions and custody level changes differs in these settings as compared to the general prison population. We also created cumulative incidence functions for the first of each of the outcomes, while accounting for competing and censoring events, among individuals entering the general prison population from the RDU and RHCP.

In a supplementary analysis, we compared rates of infractions (not limited to first events, but rather counting all events during pre-specified time periods) while in the general prison population and following release from RDU and RHCP. We calculated both 14-day and 30-day rate ratios, using Poisson regression with a generalized estimating equations approach.

In all analyses, to control for confounding, we used inverse probability of treatment weights, stabilized by the probability of exposure in the numerator. We identified the adjustment set a priori using a directed acyclic graph (Additional file 1: Fig. S1). Confounders, measured at the beginning of each eligible exposure period, were: age, gang affiliation, IQ, the number of days in restrictive housing up to that point per number of days incarcerated in that incarceration period, the number of guilty infractions up to that point per number of days incarcerated in that incarceration period, and mental health grade.

In our interpretation of the results, we relied on the magnitude of the point estimate and width of the corresponding 95% confidence interval, rather than p-values, as recommended by the American Statistical Association (Wasserstein and Lazar 2016).

We performed statistical analyses in SAS 9.4 (SAS Institute Inc., Cary, NC). This study was approved by the University of North Carolina at Chapel Hill’s Institutional Review Board (reference number: 21-134) and the NC DAC (reference number: HS2107-03).

## Results

The primary analytic cohort included 3128 people who contributed 897,822 person-days across 3209 incarcerations (Table 1). In the RDU-exposed group, 1225 people contributed 417,343 person-days to the analysis. In the RHCP-exposed group, 3059 people contributed 480,479 person-days. Overall, characteristics of RDU and RHCP person-time were similar. Most (68%) person-days were contributed by those 26–50 years old and by non-Hispanic Black men (73%). Ninety five percent of person-days were contributed by individuals who had no mental health treatment needs (i.e., M-grade 1), and most of the sample had substance use-related treatment needs (87%). The sample had spent a mean of 1683 days incarcerated (median: 1056 days). Additionally, the cohort had spent a large proportion of their incarcerated time in restrictive housing (mean: 52% or 921 days; median: 39% or 333 days). Specifically, about 22% of their incarceration was spent in RHCP, on average (median: 13%).

**Table 1:** Characteristics of placements in rehabilitative diversion units (RDU) and restrictive housing for control purposes (RHCP) among men eligible for an RDU in North Carolina prisons, 2016–2020

	Total	Restrictive housing for control purposes	Rehabilitative diversion unit
Number of people	3128	3059	1225
Number of incarcerations	3209	3139	1225
Total days contributed to analyses	897,822	480,479	417,343
	% of person-days		
*Age, years*^a^			
21–25	29.3	28.7	29.9
26–50	68.2	67.2	69.3
51+	2.6	4.0	0.9
*Race and ethnicity*^b^			
White, non-Hispanic	21.2	24.0	18.0
Black, non-Hispanic	72.6	70.6	74.9
Hispanic	3.3	2.9	3.9
Others	2.9	2.6	3.3
*Self-report, individual socioeconomic status*^b^			
High income	0.8	0.7	1.0
Middle income	40.1	38.6	41.9
Low income	47.8	49.4	46.0
Poverty	11.2	11.3	11.1
*Employment at arrest*^b^			
Employed	36.4	37.7	34.9
Unemployed	63.6	62.3	65.1
*Highest level of education completed*^b^			
< 12 years	84.2	83.2	85.2
12 years	15.8	16.7	14.7
13–15 years	0.1	0.0	0.1
*Substance use-related treatment recommendation*^b^			
None	13.2	13.7	12.6
Education	16.9	16.7	17.1
Intermediate or Intermediate/long-term	53.5	53.5	53.5
Long term	16.5	16.2	16.8
*Gang affiliation*^c^			
None	64.8	66.4	63.1
Validated 1	1.3	1.8	0.9
Validated 2	0.3	0.4	0.3
Validated 3	33.5	31.5	35.8
*Mental health grade*^a^			
1	94.5	93.0	96.2
2	5.5	7.1	3.8
*Custody level*^a^			
Close	97.3	95.0	100.0
Medium	2.4	4.5	0.0
Minimum I	0.3	0.6	0.0
	*Mean (median, 25th percentile, 75th percentile)*		
Days incarcerated^a^	1683.1 (1056.0, 427.0, 2316.0)	1626.6 (928.0, 304.0, 2196.0)	1748.2 (1180.0, 562.0, 2433.0)
Number of previous incarcerations^b^	1.9 (1.0, 0.0, 3.0)	2.1 (1.0, 0.0, 3.0)	1.7 (1.0, 0.0, 3.0)
Number of infractions/100 days incarcerated^a^	1.7 (1.2, 0.8, 2.0)	2.0 (1.3, 0.8, 2.3)	1.4 (1.1, 0.7, 1.8)
Days in any restrictive housing^a^	921.4 (333.0, 112.0, 913.0)	1014.7 (285.0, 68.0, 856.0)	813.9 (393.0, 152.0, 984.0)
Days in any restrictive housing/100 days incarcerated^a^	51.6 (38.5, 21.3, 61.8)	57.6 (38.0, 0.0, 63.9)	44.6 (38.9, 23.4, 59.3)
Days in restrictive housing for control purposes^a^	407.3 (124.0, 0.0, 434.0)	410.2 (57.0, 0.0, 370.0)	404.0 (153.0, 58.0, 469.0)
Days in restrictive housing for control purposes/100 days incarcerated^a^	22.6 (13.1, 0.0, 30.9)	21.9 (4.4, 0.0, 27.4)	23.5 (18.0, 8.4, 34.2)

^a^Calculated at the beginning of this eligibility period, during this incarceration

^b^Measured at the beginning of this incarceration

^c^The highest level of gang affiliation recorded in the prison record during this incarceration, taken at the beginning of the eligibility period. The lowest level of gang affiliation, called “affiliate,” is not represented here

Comparing rates of infractions during RDU to rates of infractions during RHCP, we found mixed results, depending on the type of infraction (Table 2). In adjusted analyses, men in RDU had higher rates of overall infractions (adjusted rate ratio (aRR): 1.6; 95% confidence interval (CI): 1.3, 1.9) than men in RHCP. However, violent infractions, the main target of the RDU program, were reduced in RDU compared to RHCP (aRR: 0.6; 95% CI: 0.4, 1.1). Adjusted rates of all other categories of infractions were higher during RDU than during RHCP assignment, particularly drug-related infractions (aRR: 2.1; 95% CI: 1.3, 3.4) and the less severe C-level infractions (aRR: 2.3; 95% CI: 1.6, 3.2).

**Table 2:** Rate ratios comparing infractions, by type, among people in a rehabilitative diversion unit (RDU), as compared to people in restrictive housing for control purposes (while eligible for an RDU), North Carolina prisons, 2016–2020

	Rate/10,000 person-days	Unadjusted rate ratio (95% CI)	Adjusted rate ratio^a^ (95% CI)
*Violent infractions*^b^			
RDU	0.6 (0.4, 0.9)	0.6 (0.3, 1.0)	0.6 (0.4, 1.1)
RHCP	1.0 (0.7, 1.5)	Ref	Ref
*Alt-violent infraction*^c^			
RDU	2.6 (2.0, 3.4)	1.1 (0.7, 1.6)	1.3 (0.9, 2.0)
RHCP	2.4 (1.8, 3.2)	Ref	Ref
*Drug-related infraction*^d^			
RDU	1.5 (1.1, 2.0)	2.0 (1.2, 3.2)	2.1 (1.3, 3.4)
RHCP	0.7 (0.5, 1.1)	Ref	Ref
*Any infraction*			
RDU	36.3 (32.1, 41.0)	1.3 (1.1, 1.6)	1.6 (1.3, 1.9)
RHCP	27.1 (23.8, 30.8)	Ref	Ref
*A-level infractions*^e^			
RDU	10.0 (8.7, 11.5)	1.3 (1.1, 1.6)	1.5 (1.2, 1.8)
RHCP	7.6 (6.6, 8.8)	Ref	Ref
*B-level infractions*^f^			
RDU	21.8 (18.9, 25.1)	1.3 (1.0, 1.6)	1.6 (1.3, 1.9)
RHCP	17.0 (14.6, 19.9)	Ref	Ref
*C-level infractions*^g^			
RDU	4.5 (3.6, 5.6)	1.8 (1.3, 2.6)	2.3 (1.6, 3.2)
RHCP	2.5 (1.9, 3.3)	Ref	Ref

*CI* Confidence interval, *RDU* rehabilitative diversion unit, *RHCP* restrictive housing for control

^a^Adjusted for age, gang affiliation, IQ, the number of days they had been in restrictive housing up to that point/days incarcerated that incarceration, the number of guilty infractions up to that point/days incarcerated that incarceration, and their mental health grade

^b^Violent infractions are not an official NC DAC categorization. Violent infractions include assault and rioting

^c^Alt-violent infractions are not an official NC DAC categorization. Alt-violent infractions are infractions that indicate a potential for violence. These include threatening to harm staff and assault with a low potential for injury

^d^Drug-related infractions are substance possession or refusing to submit to a drug or breath test

^e^A-level infractions include gang involvement, possession of a weapon to aid in assault, insurrection, riot, setting a fire, assaulting staff, and substance possession

^f^B-level infractions include disobeying an order, lock tampering, use of profane language, and threatening to harm staff

^g^C-level infractions include unauthorized use of phones or mail, possession of contraband not intended for escape or violence, creating an offensive condition, or bartering or loaning money

The secondary analytic cohort included 2227 men, of whom 2156 contributed person-days after RHCP and 679 contributed person-days after RDU (Additional file 1: Table S1). These groups contributed 49,683 and 16,303 person-days, respectively, in the analysis for a total of 65,986 person-days followed in the general prison population.

**Fig. 1:**
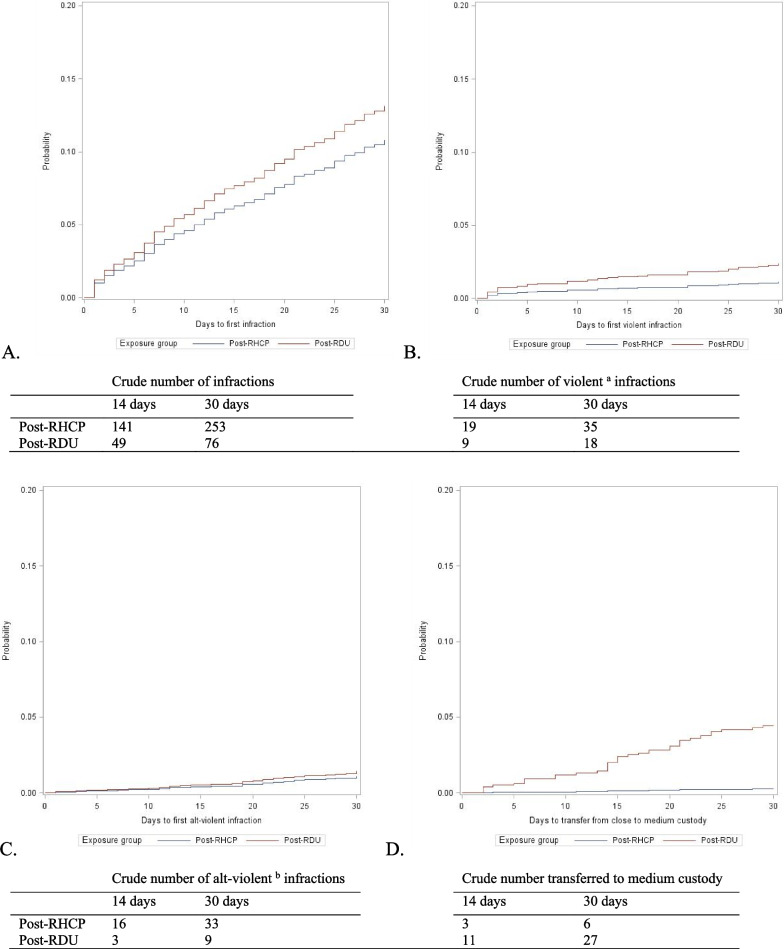
Weighted cumulative incidence functions of time to first event among people entering the general prison population from restrictive housing for control purposes and Rehabilitative Diversion Unit (RDU). Events are all infractions (Panel **A**), violent infractions (**B**), alt-violent infractions (**C**), and drug-related infractions (**D**). Note. Cumulative incidence functions are weighted using inverse probability of treatment weights (IPTW) accounting for the following confounding variables: age, gang affiliation, IQ, the number of days they had been in restrictive housing up to that point/days incarcerated that incarceration, the number of guilty infractions up to that point/days incarcerated that incarceration, and their mental health grade. RHCP, restrictive housing for control purposes; RDU, Rehabilitative Diversion Unit. ^a^Violent infractions are not an official NC DAC categorization. Violent infractions include assault and rioting. ^b^Alt-violent infractions are not an official NC DAC categorization. Alt-violent infractions are infractions that indicate a potential for violence. These include threatening to harm staff and assault with a low potential for injury

Overall, the mean days to first infraction following release into the general prison population were shorter post-RDU (11 days) than post-RHCP (14 days) (Table 3; Fig. 1). The corresponding adjusted hazard ratio estimating the hazard of infractions in the RDU group, as compared to the RHCP group, following release back to the general prison population was (adjusted hazard ratio (aHR): 1.2; 95% CI: 1.0, 1.6). For most categories of infractions, there were no differences in the hazard of first infraction between the two groups (Table 3; Fig. 1; Additional file 1: Fig. S2). However, the hazard of violent infractions post-RDU, the key focus of the RDU program, was 2.1 (95% CI: 1.1, 4.0) times the hazard post-RHCP. Adjusted analyses also indicated a higher hazard of B-level infractions post-RDU, as compared to post-RHCP (aHR: 1.4; 95% CI: 1.0, 1.9).

**Table 3:** Hazard ratios for association of Rehabilitative Diversion Unit (RDU) completion, compared to restrictive housing for control purposes completion, and first of each outcome within 30 days of exit from an RDU or restrictive housing for control purposes, North Carolina prisons, 2016–2020

	Number of first events	Mean (IQR) days until first event	Unadjusted HR (95% CI)	Adjusted HR^a^ (95% CI)
*Violent infractions*^b^				
Post-RDU	14	11.4 (1.0, 21.0)	2.0 (1.1, 3.8)	2.1 (1.1, 4.0)
Post-RHCP	26	12.4 (2.0, 21.0)	Ref	Ref
*Alt-violent infractions*^c^				
Post-RDU	8	15.4 (4.0, 24.5)	1.2 (0.5, 2.6)	1.3 (0.6, 2.8)
Post-RHCP	25	18.1 (12.0, 24.0)	Ref	Ref
*Drug-related infractions*^d^				
Post-RDU	8	11.8 (2.0, 21.0)	1.0 (0.4, 2.1)	1.0 (0.5, 2.2)
Post-RHCP	31	11.6 (5.0, 20.0)	Ref	Ref
*Any infractions*				
Post-RDU	76	11.4 (1.0, 20.0)	1.2 (0.9, 1.5)	1.2 (1.0, 1.6)
Post-RHCP	253	13.8 (6.0, 21.0)	Ref	Ref
*A-level infractions*^e^				
Post-RDU	38	11.0 (1.0, 21.0)	1.1 (0.8, 1.6)	1.2 (0.8, 1.7)
Post-RHCP	129	13.6 (6.0, 21.0)	Ref	Ref
*B-level infractions*^f^				
Post-RDU	47	12.4 (2.0, 21.0)	1.3 (0.9, 1.8)	1.4 (1.0, 1.9)
Post-RHCP	142	14.3 (6.0, 22.0)	Ref	Ref
*C-level infractions*^g^				
Post-RDU	6	9.5 (1.0, 14.0)	0.6 (0.3, 1.4)	0.7 (0.3, 1.6)
Post-RHCP	38	13.2 (7.0, 19.0)	Ref	Ref
*Movement from close to medium custody*				
Post-RDU	27	15.4 (11.0, 21.0)	17.4 (7.2, 42.1)	17.4 (7.2, 42.2)
Post-RHCP	6	16.3 (9.0, 22.0)	Ref	Ref

*CI* confidence interval, HR hazard ratio, *IQR* interquartile range, *RDU* rehabilitative diversion unit, *RHCP* restrictive housing for control purposes

^a^Adjusted for age, gang affiliation, IQ, the number of days they had been in restrictive housing up to that point/days incarcerated that incarceration, the number of guilty infractions up to that point/days incarcerated that incarceration, and their mental health grade

^b^Violent infractions are not an official NC DAC categorization. Violent infractions include assault and rioting

^c^Alt-violent infractions are not an official NC DAC categorization. Alt-violent infractions are infractions that indicate a potential for violence. These include threatening to harm staff and assault with a low potential for injury

^d^Drug-related infractions are substance possession or refusing to submit to a drug or breath test

^e^A-level infractions include gang involvement, possession of a weapon to aid in assault, insurrection, riot, setting a fire, assaulting staff, and substance possession

^g^B-level infractions include disobeying an order, lock tampering, use of profane language, and threatening to harm staff

^g^C-level infractions include unauthorized use of phones or mail, possession of contraband not intended for escape or violence, creating an offensive condition, or bartering or loaning money

During the first 30 days in the general prison population, individuals leaving RDU were more likely to be promoted to medium custody than individuals leaving RHCP (number of promotions = 27 post-RDU vs. 6 post-RHCP) (Table 3; Fig. 1). The corresponding aHR was 17.4 (95% CI: 7.2, 42.4).

Finally, in supplementary analyses, we found the 14-day and 30-day rate ratios of infractions (that included all infractions during these time periods, instead of just first events) post-RDU vs. post-RHCP were similar to hazard ratio estimates (Additional file 1: Table S2).

## Discussion

During the study period, from June 2016 to February 2020, there were 3128 incarcerated men who were eligible for RDU. Of these, 1225 ever enrolled in the program. Men eligible for RDU spent, on average, 20% of their incarceration in RHCP and an additional 30% in other forms of restrictive housing. There is a need for novel interventions to reduce repeated exposures to restrictive housing, particularly RHCP, given research showing severe adverse effects of restrictive housing exposure (Ahalt et al. 2017; Cloud et al. 2015).

RDU is an innovative intervention, intended to reduce violence and repeated cycling of men through RHCP. We found that men in RDU settings had a lower rate of violent infractions than RDU-eligible men in an RHCP environment. With respect to this core outcome, our results indicate that the RDU program is effective in reducing violence while men are enrolled in the program. However, we found this effect was not sustained once individuals were released into the general prison population. In fact, individuals returning to the general prison population from RDU had a higher hazard of first violent infraction than their peers returning from RHCP. This sort of rebound effect has been observed after other restrictive housing diversion programs in NC prisons and suggests a potential need for sustained programming beyond the resource-intensive RDU diversion program and into the general prison population (Remch et al. 2022). In addition, this highlights the need to consider the general prison environment and the contextual and situational factors that may contribute to violent behaviors in this environment, such as inconsistent application of rules and consequences for specific behaviors (McCorkle et al. 1995; Mcguire 2018; Steiner et al. 2014; Steiner and Wooldredge 2008). Of note, individuals with a history of violence in prison are given priority placement in RDU. Therefore, the higher hazard of first violent infraction after RDU compared to after RHCP might also be fully or partially attributed to uncontrolled confounding related to underlying tendency for violent behavior.

While current enrollment in the RDU was effective in reducing violent infractions, RDU enrollment did not reduce all types of infractions. Rates of drug-related infractions were notably higher during RDU than RHCP, presumably because of increased opportunity to obtain illicit drugs through increased interactions with other incarcerated men and staff.

Relatedly, by NC DAC assessment, at the beginning of their incarceration, more than half of our primary analytic sample was in need of intermediate or long-term substance use disorder treatment. We found that drug-related infractions were even more common during RDU than violent infractions. As currently designed, RDU is intended to reduce violence. Given high levels of substance use and drug-related infractions, expanding programming to include access to evidence-based substance use disorder treatment among those in these programs, as well as across the NC DAC prison population more broadly, is a critical need. At the time we conducted this evaluation, there was no formal mechanism in the RDU to treat substance use disorders. Since then, the RDU has expanded to include substance use disorder workbooks and educational materials. Given the prevalence of opioid use disorder among all incarcerated people and high risk of mortality from opioid overdose after prison release (Bukten et al. 2017; Keen et al. 2020), expansion of evidence-based treatment (i.e., medication for opioid use disorder) and harm reduction options (e.g., naloxone provision, connection to health and social services) during incarceration, including during RDU enrollment and RHCP placement, as well as prior to prison release, are also critically needed.

Our findings indicate that individuals leaving RDU are more likely to be promoted to medium custody than individuals leaving RHCP. Given known associations between restrictive prison environments and deteriorations in physical and mental health (James and Vanko 2021; Luigi et al. 2020; Strong et al. 2020), RDU as opposed to RHCP placement may have health benefits. Additionally, movement from close custody to medium custody has implications for infraction opportunity, which may inform interpretation of our findings. As opposed to close custody where individuals are housed in single-cell pods, primarily experiencing cohort-style movement with limited access to large groups or more expansive programming opportunities, medium custody provides more of these opportunities. Individuals live and interact primarily in open dormitory-style housing with greater outdoor recreation access, typically interacting daily with larger groups of individuals, both incarcerated and staff. While individuals assigned to medium custody as opposed to close are presumably considered lower risk in terms of violence or other significant infraction-incurring behavior, medium custody inherently brings an increased opportunity to engage in such activity given the added access to others, greater degree of freedom of movement, and lower level of staff supervision.

A systematic review of the potential for prison programming to reduce institutional violence found that behavioral programs have a greater effect than non-behavioral or educational/vocational programs, but overall results regarding program effectiveness are mixed (Auty et al. 2017). Like many programs evaluated, the RDU program combines multiple approaches to violence prevention (Auty et al. 2017). Specifically, the RDU program combines cognitive-behavioral approaches with social learning and education (e.g., working toward a high school equivalency degree (HiSET)). Notably, separate housing for individuals in the treatment program vs. general prison population, as is the case for the RDU program, has been demonstrated to be a positive characteristic of effective programs (Auty et al. 2017). Prior research also suggests that the implementation of a therapeutic peer community (e.g., in the case of a peer group of individuals receiving substance use disorder treatment) may also be effective for reducing institutional violence (Auty et al. 2017). As such, sustained RDU program benefits might occur with efforts to integrate established peer communities into the general prison environment. This might ease the transition from RDU to the general prison population and help reduce institutional violence overall. Additional work hypothesizes that violence prevention efforts in high-risk sectors, such as prisons, may be most effective if these efforts focus on addressing violent and disruptive behaviors of incarcerated individuals as well as the larger prison environment, including staff- and management-level factors, in which these behaviors occur (Andersen et al. 2023; Jaspers et al. 2019).

A recent scoping review of prison violence intervention efforts included ten studies of individual level interventions (Day et al. 2022). Five of these studies evaluated programs, like RDU, that were delivered to people housed separately from the general prison population. Across studies, definitions and timing of violence outcomes varied. Findings from these studies were mixed, with some finding program effects on violence reduction during or after the intervention and others finding no program effects on violence. Day et al. (2022) concluded that despite the clear need for violence prevention work in prisons, empirical research on programmatic interventions is rare. The RDU evaluation presented here adds to this important but small literature.

## Limitations

Through our inclusion criteria and inverse probability of treatment weights, we aimed to create exchangeability (i.e., balance of important potential confounders) between the RDU and RHCP groups. However, we may not have successfully accounted for all the complex factors that impact selection into the RDU, including propensity toward violence. Additionally, because of the structure of the environments and opportunity to engage with other incarcerated persons, there may be different opportunity for infractions during RDU and RHCP which would affect the comparison of infraction rates in these environments. Furthermore, as described above, there may be different opportunity for infractions in close as opposed to medium custody, which would impact interpretation of the infractions outcomes while individuals were in the general prison population. Finally, some individuals do not go directly from RHCP to the general prison population, but rather are placed in modified housing, prior to the general population. NC DAC intends for individuals with a greater history of violence to be placed in modified housing prior to the general population; however, placement in modified housing is known to be subject to bed availability and other factors. Thus, most RHCP-assigned men (approximately 80%) transitioned directly to the general population. As we omitted individuals from the second analysis who were enrolled in modified housing directly after RDU or RHCP, we may have excluded individuals with a greater history of and tendency toward violence. Still, this analysis includes a detailed examination of critical safety outcomes experienced both during and immediately following release from an innovative prison violence prevention program, as compared to a commonly used pathway (i.e., RHCP), using robust methods to remove measurable bias.

## Conclusions

There is a sizable population of men in NC prisons who spend a large proportion of their incarceration in restrictive housing. The RDU is one of a few programs implemented in NC prisons to reduce the frequent cycling of individuals through restrictive housing. We found that the RDU is effective in reducing violent infractions during the program, one of its main objectives. However, drug-related infractions were common in the RDU. Since the time of our evaluation, the RDU has expanded to include some substance use disorder educational materials for a substantial number of program participants. Further evaluation of the effectiveness of these materials is warranted, as is critical consideration of alignment with best practice treatment guidelines. Perhaps the most impactful consequence of RDU placement was that it resulted in more individuals being promoted to medium custody following program completion, which is known to be associated with improved physical and mental health. Although other benefits may not be sustained after RDU completion, the increased promotion to medium custody alone indicates that the RDU may be a promising prison program from a health perspective. To sustain potential benefits of the RDU, as compared to a more restrictive environment, sustained programming (e.g., therapeutic peer communities) may be needed to continue to support individuals following the intensive service environment of the RDU and while in the general prison population.

## Supplementary Information

**Additional file 1**. Supplementary Material.

## Data Availability

Data used in this study were shared with the authors from North Carolina Department of Adult Correction, and the authors do not have permission to share these data.
