# Supplementary material for: Evaluation of a prison violence prevention program: impacts on violent and non-violent prison infractions
Source: Inj Epidemiol. 2023 Jul 24;10:36. doi: 10.1186/s40621-023-00450-9 (PMC10367332; doi:10.1186/s40621-023-00450-9)
Supplement: Supplementary file 1 — Additional file 1. Supplementary Material. [file 40621_2023_450_MOESM1_ESM.docx]

SUPPLEMENT.

**Supplemental Figure S1.** Directed acyclic graph used for identification of adjustment set. Adjustment set indicated by white circles. Causal path highlighted in green.


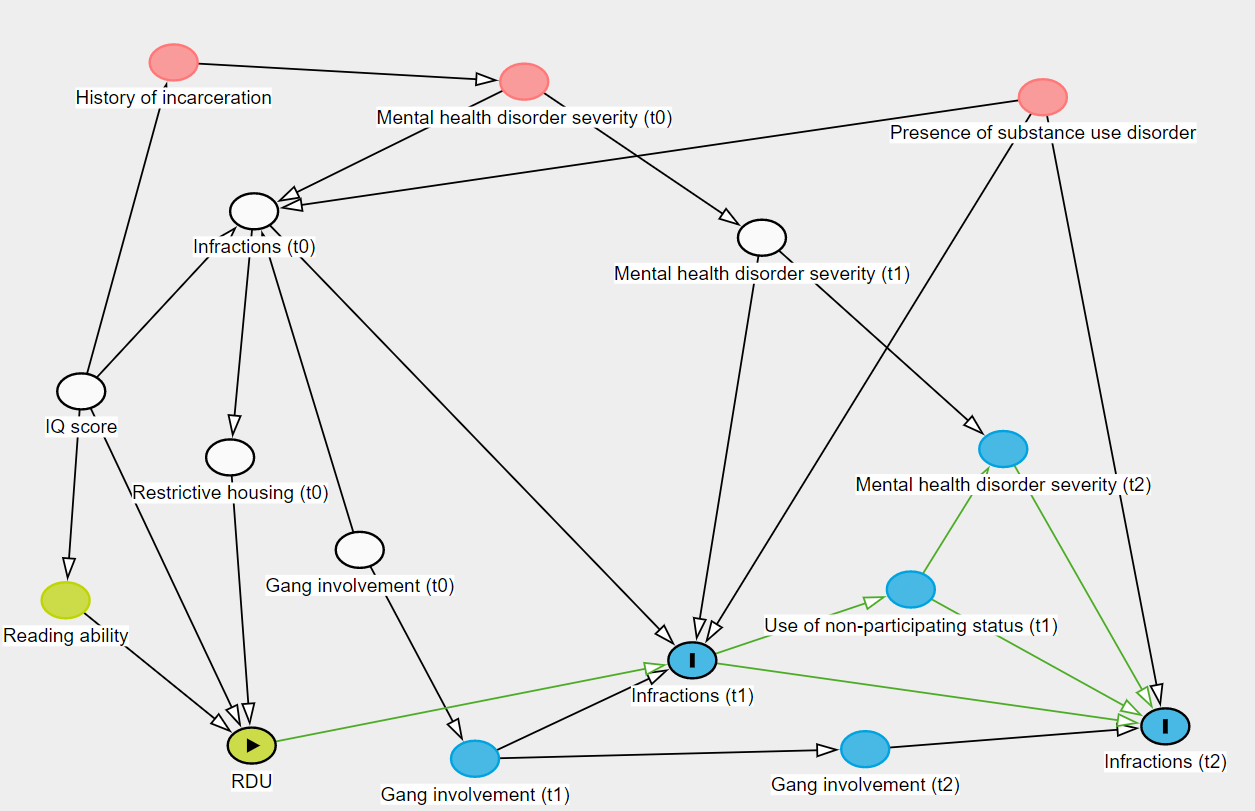


We represent the data at three time points, labelled as t0, t1, and t2. These are prior to RDU exposure, during RDU exposure, and the end of RDU exposure, respectively. Covariates used in the adjustment set are measured at t0 and outcomes are measured through t1 and t2.

**Supplemental Table S1.** Characteristics of cohorts following release from rehabilitative diversion units (RDU) and restrictive housing for control purposes (RHCP) among men who had been eligible for an RDU in North Carolina prisons, 2016- 2020

|  | | Total | Restrictive Housing for Control Purposes | Rehabilitative Diversion Unit |
| --- | --- | --- | --- | --- |
| Number of people | | 2,227 | 2,156 | 679 |
| Number of incarcerations | | 2,230 | 2,159 | 679 |
| Total days contributed to analyses | | 65,986 | 49,683 | 16,303 |
|  | | % of person-days | | |
| Age, years ^a^ | |  |  |  |
|  | 21-25 | 29.7 | 28.7 | 28.7 |
|  | 26-50 | 69.1 | 68.7 | 70.2 |
|  | 51+ | 2.2 | 2.6 | 1.1 |
| Race and ethnicity ^b^ | |  |  |  |
|  | White, non-Hispanic | 20.9 | 22.6 | 15.6 |
|  | Black, non-Hispanic | 73.01 | 71.3 | 78.3 |
|  | Hispanic | 3.2 | 3.1 | 3.4 |
|  | Others | 2.9 | 2.9 | 2.7 |
| Self-report individual socioeconomic status ^b^ | |  |  |  |
|  | High income | 1.0 | 0.9 | 1.2 |
|  | Middle income | 39.9 | 39.2 | 42.1 |
|  | Low income | 48.0 | 48.2 | 47.5 |
|  | Poverty | 11.1 | 11.8 | 9.3 |
|  | Missing |  |  |  |
| Employment at arrest ^b^ | |  |  |  |
|  | Employed | 37.2 | 38.6 | 32.6 |
|  | Unemployed | 62.9 | 61.4 | 67.4 |
|  | Missing |  |  |  |
| Highest level of education completed ^b^ | |  |  |  |
|  | < 12 years | 83.2 | 82.6 | 84.8 |
|  | 12 years | 16.7 | 17.3 | 15.1 |
|  | 13-15 years | 0.1 | 0.1 | 0.2 |
| Substance use-related treatment recommendation ^b^ | |  |  |  |
|  | None | 13.4 | 13.5 | 13.3 |
|  | Education | 17.0 | 16.5 | 18.4 |
|  | Intermediate or Intermediate/Long-Term | 54.5 | 54.6 | 54.0 |
|  | Long-Term | 15.1 | 15.4 | 14.3 |
|  | Missing |  |  |  |
| Gang affiliation ^c^ | |  |  |  |
|  | None | 64.9 | 65.1 | 64.3 |
|  | Validated 1 | 1.6 | 1.9 | 0.8 |
|  | Validated 2 | 0.3 | 0.4 | 0.2 |
|  | Validated 3 | 33.2 | 32.6 | 34.8 |
| Mental health grade ^a^ | |  |  |  |
|  | 1 | 95.5 | 94.9 | 97.3 |
|  | 2 | 4.5 | 5.1 | 2.7 |
| Custody level ^a^ | |  |  |  |
|  | Close | 96.2 | 94.9 | 100.0 |
|  | Medium | 3.2 | 4.3 | 0.0 |
|  | Minimum I | 0.6 | 0.8 | 0.0 |
|  | Missing |  |  |  |
|  | | Mean (median, 25^th^ percentile, 75^th^ percentile) | | |
| Days incarcerated ^a^ | | 1622.9 (989.0, 407.0, 2,160.0) | 1591.7 (937.0, 359.0, 2099.0) | 1718.0 (1150.0, 565.0, 2370.0) |
| Number of previous incarcerations ^b^ | | 1.9 (1.0, 0.0, 3.0) | 1.9 (1.0, 0.0, 3.0) | 1.6 (1.0, 0.0, 3.0) |
| Number of infractions / 100 days incarcerated ^a^ | | 1.6 (1.2, 0.7, 1.9) | 1.7 (1.2, 0.7, 2.0) | 1.3 (1.1, 0.7, 1.7) |
| Days in any restrictive housing ^a^ | | 718.1 (283.0, 85.00, 778.0) | 711.6 (254.0, 64.0, 721.0) | 737.9 (370.0, 151.0, 926.0) |
| Days in any restrictive housing / 100 days incarcerated ^a^ | | 51.7 (33.3, 17.2, 55.5) | 54.5 (32.1, 15.7, 55.0) | 42.9 (37.5, 22.7, 56.9) |
| Days in restrictive housing for control purposes ^a^ | | 329.4 (74.0, 0.0, 339.0) | 315.4 (0.0, 0.0, 314.0) | 372.1 (134.0, 57.0, 434.0) |
| Days in restrictive housing for control purposes / 100 days incarcerated ^a^ | | 24.3 (8.2, 0.0, 25.4) | 25.0 (0.0, 0.0, 21.4) | 22.4 (16.0, 8.1, 32.8) |

^a^ Calculated at the beginning of this eligibility period, during this incarceration

^b^ Measured at the beginning of this incarceration

^c^ The highest level of gang affiliation recorded in the prison record during this incarceration taken at the beginning of the eligibility period. The lowest level of gang affiliation, called “affiliate” is not represented here

A.
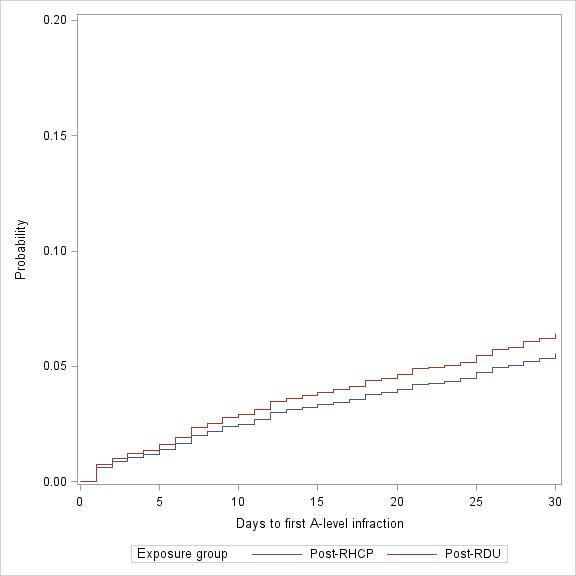
 B.
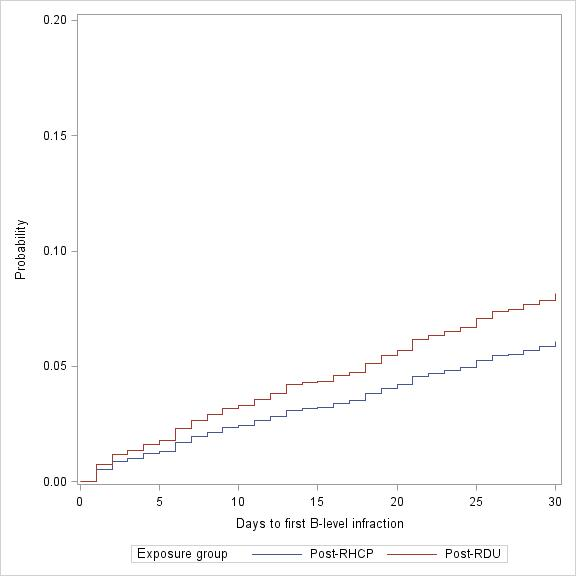


|  | Crude number of A-level ^a^ infractions | |  | Crude number of B-level ^b^ infractions | |
| --- | --- | --- | --- | --- | --- |
|  | 14 days | 30 days |  | 14 days | 30 days |
| Post-RHCP | 82 | 141 |  | 78 | 157 |
| Post-RDU | 25 | 42 |  | 31 | 49 |

C.
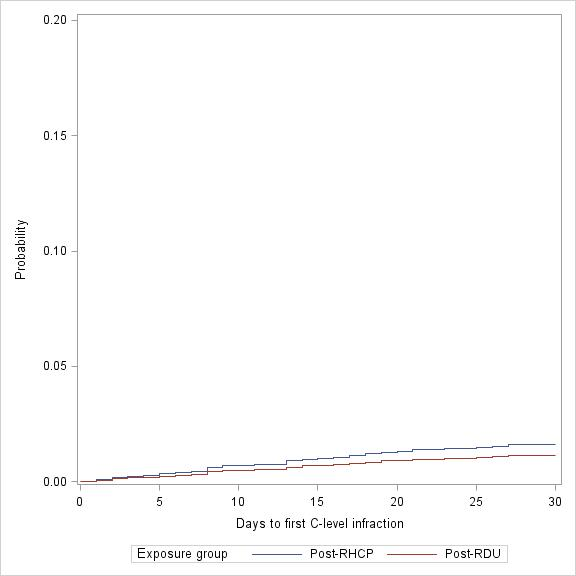
 D.
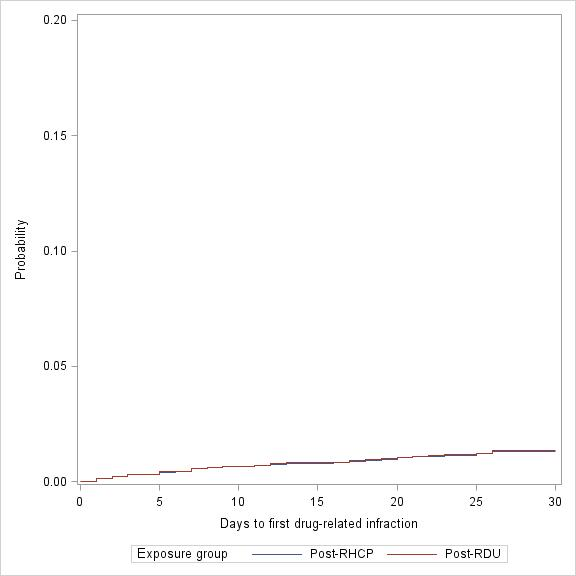


|  | Crude number of C-level ^c^ infractions | |  | Crude number of drug-related infractions | |
| --- | --- | --- | --- | --- | --- |
|  | 14 days | 30 days |  | 14 days | 30 days |
| Post-RHCP | 30 | 49 |  | 24 | 37 |
| Post-RDU | 5 | 6 |  | 5 | 9 |

**Supplemental Figure S2**. Weighted cumulative incidence functions of time to first event among people entering the general prison population from restrictive housing for control purposes and Rehabilitative Diversion Unit (RDU). Events are A-level infractions (Panel A), B-level infractions (B), C-level infractions (C), and drug-related infractions (D).

Note. Cumulative incidence functions are weighted using inverse probability of treatment weights (IPTW) accounting for the following confounding variables: age, gang affiliation, IQ, the number of days they had been in restrictive housing up to that point/ days incarcerated that incarceration, the number of guilty infractions up to that point/ days incarcerated that incarceration, and their mental health grade.

RHCP, restrictive housing for control purposes; RDU, Rehabilitative Diversion Unit

^a^ A-level infractions include gang involvement, possession of a weapon to aid in assault, insurrection, riot, setting a fire, assaulting staff, and substance possession.

^d^ B-level infractions include disobeying an order, lock tampering, use of profane language, and threatening to harm staff.

^c^ C-level infractions include unauthorized use of phones or mail, possession of contraband not intended for escape or violence, creating an offensive condition, or bartering or loaning money.

**Supplemental Table S2**. Rate ratios comparing infractions, by type, among people in the general prison population following rehabilitative diversion unit (RDU) release, as compared to people in the general prison population following restrictive housing for control purpose release (who were eligible for a RDU), North Carolina prisons, 2016-2020

|  | | Rate / 10,000 person-days | Unadjusted Rate Ratio  (95% CI) | Adjusted ^a^ Rate Ratio  (95% CI) |
| --- | --- | --- | --- | --- |
| Violent infractions ^b^ | |  |  |  |
|  | 14-days |  |  |  |
|  | Post-RDU | 9.6 (4.8, 19.1) | 1.5 (0.7, 3.5) | 1.7 (0.7, 4.1) |
|  | Post-RHCP | 6.3 (3.9, 10.1) | Ref | Ref |
|  | 30-days |  |  |  |
|  | Post-RDU | 8.3 (4.9, 13.9) | 1.6 (0.8, 3.1) | 1.7 (0.9, 3.3) |
|  | Post-RHCP | 5.2 (3.5, 7.6) | Ref | Ref |
| Alt-violent infraction ^c^ | |  |  |  |
|  | 14-days |  |  |  |
|  | Post-RDU | 3.6 (1.2, 11.1) | 1.0 (0.3, 3.5) | 0.9 (0.3, 3.3) |
|  | Post-RHCP | 3.7 (2.0, 6.9) | Ref | Ref |
|  | 30-days |  |  |  |
|  | Post-RDU | 3.5 (1.6, 7.9) | 0.9 (0.4, 2.2) | 1.0 (0.4, 2.6) |
|  | Post-RHCP | 4.0 (2.6, 6.3) | Ref | Ref |
| Drug-related infraction ^d^ | |  |  |  |
|  | 14-days |  |  |  |
|  | Post-RDU | 4.8 (1.8, 12.7) | 0.6 (0.2, 1.8) | 0.7 (0.2, 2.1) |
|  | Post-RHCP | 7.8 (4.8, 12.6) | Ref | Ref |
|  | 30-days |  |  |  |
|  | Post-RDU | 3.5 (1.6, 7.9) | 0.6 (0.2, 1.4) | 0.6 (0.3, 1.6) |
|  | Post-RHCP | 6.0 (4.1, 8.7) | Ref | Ref |
| Any infraction | |  |  |  |
|  | 14-days |  |  |  |
|  | Post-RDU | 80.0 (58.8, 108.9) | 1.1 (0.7, 1.6) | 1.2 (0.8, 1.7) |
|  | Post-RHCP | 72.2 (56.4, 92.4) | Ref | Ref |
|  | 30-days |  |  |  |
|  | Post-RDU | 60.2 (46.8, 77.5) | 0.8 (0.6, 1.1) | 0.9 (0.6, 1.2) |
|  | Post-RHCP | 72.8 (60.2, 88.1) | Ref | Ref |
| A-level infractions ^e^ | |  |  |  |
|  | 14-days |  |  |  |
|  | Post-RDU | 28.7 (18.6, 44.1) | 0.9 (0.6, 1.5) | 1.0 (0.6, 1.6) |
|  | Post-RHCP | 31.1 (23.8, 40.5) | Ref | Ref |
|  | 30-days |  |  |  |
|  | Post-RDU | 22.4 (15.9, 31.8) | 0.8 (0.6, 1.3) | 0.9 (0.6, 1.3) |
|  | Post-RHCP | 26.7 (21.8, 32.8) | Ref | Ref |
| B-level infractions ^f^ | |  |  |  |
|  | 14-days |  |  |  |
|  | Post-RDU | 44.2 (29.5, 66.2) | 1.3 (0.7, 2.2) | 1.3 (0.8, 2.3) |
|  | Post-RHCP | 34.4 (24.2, 49.0) | Ref | Ref |
|  | 30-days |  |  |  |
|  | Post-RDU | 33.1 (23.9, 45.8) | 0.8 (0.6, 1.3) | 0.9 (0.6, 1.4) |
|  | Post-RHCP | 39.2 (30.2, 50.9) | Ref | Ref |
| C-level infractions ^g^ | |  |  |  |
|  | 14-days |  |  |  |
|  | Post-RDU | 7.2 (2.8, 18.0) | 1.1 (0.4, 3.1) | 1.2 (0.4, 3.5) |
|  | Post-RHCP | 6.7 (3.9, 11.3) | Ref | Ref |
|  | 30-days |  |  |  |
|  | Post-RDU | 4.7 (2.0, 11.0) | 0.7 (0.3, 1.7) | 0.8 (0.3, 2.1) |
|  | Post-RHCP | 6.9 (4.8, 10.1) | Ref | Ref |

CI: Confidence interval

RDU: Rehabilitative Diversion Unit

RHCP: Restrictive housing for control

^a^ Adjusted for age, gang affiliation, IQ, the number of days they had been in restrictive housing up to that point/ days incarcerated that incarceration, the number of guilty infractions up to that point/ days incarcerated that incarceration, and their mental health grade.

^b^ Violent infractions are not an official NC DAC categorization. Violent infractions include assault and rioting.

^c^ Alt-violent infractions are not an official NC DAC categorization. Alt-violent infractions are infractions that indicate a potential for violence. These include threatening to harm staff and assault with a low potential for injury.

^d^ Drug-related infractions are substance possession or refusing to submit to a drug or breath test.

^e^ A-level infractions include gang involvement, possession of a weapon to aid in assault, insurrection, riot, setting a fire, assaulting staff, and substance possession.

^g^ B-level infractions include disobeying an order, lock tampering, use of profane language, and threatening to harm staff.

^g^ C-level infractions include unauthorized use of phones or mail, possession of contraband not intended for escape or violence, creating an offensive condition, or bartering or loaning money.
